# Supplementary material for: Assigning Defined Daily/Course Doses for Antimicrobials in Turkeys to Enable a Cross-Country Quantification and Comparison of Antimicrobial Use
Source: Antibiotics (Basel). 2021 Aug 12;10(8):971. doi: 10.3390/antibiotics10080971 (PMC8388960; doi:10.3390/antibiotics10080971)
Supplement: Supplementary file 1 [file antibiotics-10-00971-s001.zip › antibiotics-1326495-supplementary.pdf]

Supplementary Materials Part A: Results on DDD_{turkey} DCD_{turkey} and antimicrobial usage (not shown in the main text)

Table S1. Number of products registered for use in turkeys in Germany (DE), France (FR) and Spain (ES). Stratified by unique combination of antimicrobial class and administration route.

Antimicrobial class	Feed				Injection				Water				Total			
	Country															
	DE	FR	ES	ALL	DE	FR	ES	ALL	DE	FR	ES	ALL	DE	FR	ES	ALL
**Aminoglycosides***	-	-	-	-	-	-	-	-	4	2	3	9	4	2	3	9
[1]BLS penicillins	-	-	-	-	-	-	-	-	3	1	2	6	3	1	2	6
**[2]ES penicillins***	-	-	-	-	-	1	-	1	7	14	8	29	7	15	8	30
**Fluoroquinolones***	-	-	-	-	-	-	-	-	11	12	16	39	11	12	16	39
**[3]Linco/Spec**	-	-	-	-	-	2	-	2	2	-	-	2	2	2	-	4
**Macrolides***	-	6	-	6	-	-	-	-	8	12	8	28	8	18	8	34
Pleuromutilins	1	3	-	4	-	-	-	-	6	6	-	12	7	9	-	16
**Polymyxins***	-	3	-	3	-	2	-	2	5	17	9	31	5	22	9	36
**Tetracyclines**	-	3	6	9	-	1	-	1	14	27	14	55	14	31	20	65
**[4]Trim/Sulfa**	-	3	-	3	-	-	-	-	-	13	-	13	-	16	-	16
Total	1	18	6	25	-	6	-	6	60	104	60	224	61	128	66	255
[5]Human CIA*	-	9	-	9	-	3	-	3	31	55	41	127	31	67	41	139
**[6]Vet CIA**	-	15	6	21	-	6	-	6	51	97	58	206	51	118	64	233

[1]BLS penicillins = β-Lactamase-Sensitive penicillins; [2]ES penicillins = Extended Spectrum penicillins; [3]Linco/Spec = lincomycin in combination with spectinomycin; [4]Trim/Sulfa = trimethoprim in combination with a sulfonamide. [5]Human CIA = critically important antimicrobials for human medicine as determined by WHO [46], antimicrobial classes that belong to this group are indicated with an asterisk (*). [6]Vet CIA = critically important antimicrobials for veterinary medicine as determined by OIE [47], antimicrobial classes that belong to this group are indicated in **bold**.

Table S2. List of the mean consensus DDD_{turkey} (defined daily dose turkey) per active substance and administration route established with data from three European Union countries. The median, mode, minimum and maximum recommended dosages are also provided. The percentage of products that have a recommended daily dosage that differs plus or minus 10% from the mean consensus DDD_{turkey} were listed, as well as the difference (in percentage) between the highest and lowest recommended daily dosage. The last column describes the DCD_{turkey} (defined course dose turkey) established using the mean consensus DDD_{turkey} and the mean consensus treatment duration for the specific active substances and administration routes. DDDA stands for defined daily dose animal and is the same as DDD_{turkey}.

Active Substance (ATCvet code)	Administration Route	Total and Country	DDD_{turkey} and Long Acting Factor	Count	Mean = Consensus DDD_{turkey}	Median	Min	Max	% Products < 90% from Consensus DDD_{turkey}	% Products > 110% from Consensus DDD_{turkey}	Difference Highest vs Lowest (%)	DCD_{turkey}
Amoxicillin (QJ01CA04)	water	Total	DDDA*	25	**14.1**	**15.3**	**10.00**	**20.00**	**24.0%**	**12.0%**	**200.0%**	**60.6**
			LA Factor		1.00	1.00	1.00	1.00				

		France	DDDA	12	13.4	12.6	10.0	15.3	50.0%	50.0%	153.0%	
		Germany	DDDA	7	15.3	15.3	13.1	17.4	0.0%	0.0%	133.0%	
		Spain	DDDA	8	15.5	15.3	13.1	20.0	0.0%	13.0%	153.0%	
Ampicillin (QJ01CA01)	Water	Total	DDDA	4	**20.0**	**20.0**	**20.0**	**20.0**	**0.0%**	**0.0%**	**0.0%**	**60.0**
			LA Factor		1.0	1.0	1.0	1.0				
		France	DDDA	4	20.0	20.0	20.0	20.0	0.0%	0.0%	0.0%	
		Germany	DDDA	0	-	-	-	-	-	-	-	
		Spain	DDA	0	-	-	-	-	-	-	-	
	Parenteral	Total	DDDA	1	**10.5**	**10.5**	**7.0**	**14.0**	**0.0%**	**0.0%**	**200.0%**	**31.5**
			LA Factor		1.0	1.0	1.0	1.0				
		France	DDDA	1	10.5	10.5	7.0	14.0	0.0%	0.0%	200.0%	
		Germany	DDDA	0	-	-	-	-	-	-	-	
		Spain	DDDA	0	-	-	-	-	-	-	-	
Benzylpenicillin-Kalium (J01CE01)	Water	Total	DDDA	1	**28.8**	**28.8**	**28.8**	**28.8**	**0.0%**	**0.0%**	**0.0%**	**115**
			LA Factor		1.0	1.0	1.0	1.0				
		France	DDDA	0	-	-	-	-	-	-	-	
		Germany	DDDA	1	28.8	28.8	28.8	28.8	0.0%	0.0%	0.0%	
		Spain	DDDA	0	-	-	-	-	-	-	-	
Colistin (QA07AA10)	Feed	Total	DDDA	3	**4.7**	**5.0**	**4.0**	**5.0**	**33.3%**	**0.0%**	**125.0%**	**32.7**
			LA Factor		1.0	1.0	1.0	1.0				
		France	DDDA	3	4.7	5.0	4.0	5.0	33.3%	0.0%	125.0%	
		Germany	DDDA	0	-	-	-	-	-	-	-	
		Spain	DDDA	0	-	-	-	-	-	-	-	
	Water	Total	DDDA	31	**3.4**	**3.75**	**2.5**	**4.8**	**45.0%**	**55.0%**	**192.0%**	**13.8**
			LA Factor		1.00	1.00	1.00	1.00				
		France	DDDA	17	3.5	3.75	2.5	3.8	35.0%	0.0%	152.0%	

		Germany	DDDA	5	4.4	4.8	3.8	4.8	40.0%	0.0%	126.0%	
		Spain	DDDA	9	2.7	2.5	2.5	4.1	0.0%	11.0%	164.0%	
	Parenteral	Total	DDDA	2	**2.5**	**2.5**	**2.5**	**2.5**	**0.0%**	**0.0%**	**0.0%**	**7.5**
			LA Factor		1.00	1.00	1.00	1.00				
		France	DDDA	2	2.5	2.5	2.5	2.5	0.0%	0.0%	0.0%	
		Germany	DDDA	0	-	-	-	-	-	-	-	
		Spain	DDDA	0	-	-	-	-	-	-	-	
Doxycycline (QJ01AA02)	Water	Total	DDDA	37	**18.8**	**20.0**	**10.0**	**25.00**	**27.0%**	**32.0%**	**250.0%**	**90.4**
			LA Factor		1.00	1.00	1.00	1.00				
		France	DDDA	13	15.9	20.0	10.0	25.0	54.0%	38.0%	250.0%	
		Germany	DDDA	13	22.1	25.0	10.0	25.0	15.0%	62.0%	250.0%	
		Spain	DDDA	11	18.4	17.4	10.0	25.0	45.0%	36.0%	250.0%	
Enrofloxacin (QJ01MA90)	Water	Total	DDDA	29	**10.0**	**10.0**	**10.0**	**10.0**	**0.0%**	**0.0%**	**0.0%**	**40.0**
			LA Factor		1.00	1.00	1.00	1.00				
		France	DDDA	7	10.0	10.0	10.0	10.0	0.0%	0.0%	0.0%	
		Germany	DDDA	11	10.0	10.0	10.0	10.0	0.0%	0.0%	0.0%	
		Spain	DDDA	11	10.0	10.0	10.0	10.0	0.0%	0.0%	0.0%	
Flumequin (QJ01MB07)	Water	Total	DDDA	10	**15.0**	**15.0**	**12.0**	**24.0**	**50.0%**	**50.0%**	**200.0%**	**66.0**
			LA Factor		1.00	1.00	1.00	1.00				
		France	DDDA	5	12.0	12.0	12.0	12.0	0.0%	0.0%	0.0%	
		Germany	DDDA	0	-	-	-	-	-	-	-	
		Spain	DDDA	5	18.0	18.0	12.0	14.0	0.0%	0.0%	200.0%	
Lincomycin + Spectinomycin (QA07AA99)	Water	Total	DDDA	2	**62.5**	**62.5**	**49.5**	**75.0**	**50.0%**	**50.0%**	**152.0%**	**287.2**
			LA Factor		1.00	1.00	1.00	1.00				

		France	DDDA	0	-	-	-	-	-	-	-	
		Germany	DDDA	2	62.5	62.5	49.5	75.0	50.0%	50.0%	152.0%	
		Spain	DDDA	0	-	-	-	-	-	-	-	
	Parenteral	Total	DDDA	2	**30.0**	**30.0**	**30.0**	**30.0**	**0.0%**	**0.0%**	**0.0%**	**90.0**
			LA Factor		1.00	1.00	1.00	1.00				
		France	DDDA	2	30.0	30.0	30.0	30.0	0.0%	0.0%	0.0%	
		Germany	DDDA	0	-	-	-	-	-	-	-	
		Spain	DDDA	0	-	-	-	-	-	-	-	
Neomycin (QA07AA01)	Water	Total	DDDA	9	**22.4**	**30.00**	**5.0**	**39.9**	**44.0%**	**60.0%**	**798.0%**	**93.7**
			LA Factor		1.0	1.0	1.0	1.0				
		France	DDDA	2	16.7	16.7	11.5	20.0	50.0%	50.0%	174.0%	
		Germany	DDDA	4	30.0	30.0	30.0	30.0	0.0%	0.0%	0.0%	
		Spain	DDDA	3	16.1	7.5	5.0	39.9	66.7%	33.3%	798%	
Oxytetracycline (QJ01AA06)	Feed	Total	DDDA	9	**18.4**	**10.2**	**5.1**	**40.0**	**66.7%**	**33.3%**	**784.0%**	**180.9**
			LA Factor		1.0	1.0	1.0	1.0				
		France	DDDA	3	40.0	40.0	40.0	40.0	0.0%	0.0%	0.0%	
		Germany	DDDA	0	-	-	-	-	-	-	-	
		Spain	DDDA	6	7.6	7.6	5.1	10.2	50.0%	50.0%	200.0%	
	Water	Total	DDDA	18	**24.5**	**20.0**	**8.4**	**50.0**	**89.0%**	**11.0%**	**599.0%**	**106.7**
			LA Factor		1.0	1.0	1.0	1.0				
		France	DDDA	14	19.9	20.0	16.0	22.0	0.0%	0.0%	138.0%	
		Germany	DDDA	1	80.0	80.0	80.0	80.0	0.0%	0.0%	0.0%	
		Spain	DDDA	3	27.5	20.0	8.4	50.0	66.7%	33.3%	595.0%	
	Parenteral	Total	DDDA	1	**7.5**	**7.5**	**5.0**	**10.0**	**0.0%**	**0.0%**	**200.0%**	**30.0**

			LA Factor		1.0	1.0	1.0	1.0				
		France	DDDA	1	7.5	7.5	5.0	10.0	¨0.0%	0.0%	200.0%	
		Germany	DDDA	0	-	-	-	-	-	-	-	
		Spain	DDDA	0	-	-	-	-	-	-	-	
Phenoxymethylpenicillin (QJ01CE02)	Water	Total	DDDA	6	**16.8**	**16.8**	**16.8**	**16.8**	**0.0%**	**0.0%**	**0.0%**	**83.8**
			LA Factor		1.00	1.00	1.00	1.00				
		France	DDDA	1	16.8	16.8	16.8	16.8	0.0%	0.0%	0.0%	
		Germany	DDDA	3	16.8	16.8	16.8	16.8	0.0%	0.0%	0.0%	
		Spain	DDDA	2	16.8	16.8	16.8	16.8	0.0%	0.0%	0.0%	
Spiramycin (QJ01FA02)	Water	Total	DDDA	2	**23.25**	**23.25**	**15.5**	**31.0**	**50.0%**	**50.0%**	**200.0%**	**89.1**
			LA Factor		1.00	1.00	1.00	1.00				
		France	DDDA	2	23.25	23.25	15.5	31.0	50.0%	50.0%	200.0%	
		Germany	DDDA	0	-	-	-	-	-	-	-	
		Spain	DDDA	0	-	-	-	-	-	-	-	
Tiamulin QJ01XQ01)	Feed	Total	DDDA	4	**32.2**	**32.2**	**32.0**	32.4	**0.0%**	**0.0%**	**101.0%**	**224.8**
			LA Factor		1.00	1.00	1.00	1.00				
		France	DDDA	3	32.1	32.0	32.0	32.4	0.0%	0.0%	101.0%	
		Germany	DDDA	1	32.4	32.4	32.4	32.4	0.0%	0.0%	0.0%	
		Spain	DDDA	0	-	-	-	-	-	-	-	
	Water	Total	DDDA	12	**28.3**	**32.4**	**20.2**	**32.4**	**33.3%**	**66.7%**	**160.0%**	**130.2**
			LA Factor		1.00	1.00	1.00	1.00				
		France	DDDA	6	32.4	32.4	32.4	32.4	0.0%	0.0%	0.0%	
		Germany	DDDA	6	24.3	20.2	20.2	32.4	66.7%	33.3%	160.0%	

		Spain	DDDA	0	-	-	-	-	-	-	-	
Tilmicosin (QJ01FA91)	Water	Total	DDDA	6	**18.5**	**18.5**	**10.0**	**27.0**	**0.0%**	**0.0%**	**270.0%**	**55.5**
			LA Factor		1.00	1.00	1.00	1.00				
		France	DDDA	2	18.5	18.5	10.0	27.0	0.0%	0.0%	270.0%	
		Germany	DDDA	1	18.5	18.5	10.0	27.0	0.0%	0.0%	270.0%	
		Spain	DDDA	3	18.5	18.5	10.0	27.0	0.0%	0.0%	270.0%	
Trimethoprim + Sulfadiazine (QJ01EW10)	Feed	Total	DDDA	3	**60.0**	**60.0**	**60.0**	**60.0**	**0.0%**	**0.0%**	**0.0%**	**420.0**
			LA Factor		1.00	1.00	1.00	1.00				
		France	DDDA	3	60.0	60.0	60.0	60.0	0.0%	0.0%	0.0%	
		Germany	DDDA	0	-	-	-	-	-	-	--	
		Spain	DDDA	0	-	-	-	-	-	-	--	
	Water	Total	DDDA	5	30.7	**30.0**	**30.0**	**33.6**	**0.0%**	**0.0%**	**112.0%**	**165.6**
			LA Factor		1.00	1.00	1.00	1.00				
		France	DDDA	5	30.7	30.0	30.0	33.6	0.0%	0.0%	112.0%	
		Germany	DDDA	0	-	-	-	-	-	-	--	
		Spain	DDDA	0	-	-	-	-	-	-	--	
Trimethoprim + Sulfadimethoxine (QJ01EW09)	Water	Total	DDDA	7	**36.0**	**34.0**	**24.9**	**56.7**	**14.0%**	**14.0%**	**228.0%**	**190.3**
			LA Factor		1.00	1.00	1.00	1.00				
		France	DDDA	7	36.0	34.0	24.9	56.7	14.0%	14.0%	228.0%	
		Germany	DDDA	0	-	-	-	-	-	-	--	
		Spain	DDDA	0	-	-	-	-	-	-	--	
Trimethoprim + Sulfamethoxypyridazine (QJ01EW15)	Water	Total	DDDA	1	**28.2**	**28.2**	**28.2**	**28.2**	**0.0%**	**0.0%**	**0.0%**	**113.0**
			LA Factor		1.00	1.00	1.00	1.00				

		France	DDDA	1	28.2	28.2	28.2	28.2	0.0%	0.0%	0.0%	
		Germany	DDDA	0	-	-	-	-	-	-	--	
		Spain	DDDA	0	-	-	-	-	-	-	--	
Tylosin (QJ01FA90)	Feed	Total	DDDA	6	**75.0**	**75.0**	**75.0**	**75.0**	**0.0%**	**0.0%**	**0.0%**	**262.5**
			LA Factor		1.00	1.00	1.00	1.00				
		France	DDDA	6	75.0	75.0	75.0	75.0	0.0%	0.0%	0.0%	
		Germany	DDDA	0	-	-	-	-	-	-	--	
		Spain	DDDA	0	-	-	-	-	-	-	--	
	Water	Total	DDDA	17	**75.3**	**75.0**	**41.2**	**96.3**	**18.0%**	**41%**	**230.0%**	**280.2**
			LA Factor		1.00	1.00	1.00	1.00				
		France	DDDA	7	75.0	75.0	75.0	75.0	0.0%	0.0%	0.0%	
		Germany	DDDA	6	90.4	87.5	87.5	96.3	0.0%	0.0%	110.0%	
		Spain	DDDA	4	53.3	41.9	41.85	87.5	75.0%	25.0%	209.0%	
Tylvalosin (QJ01FA92)	Water	Total	DDDA	3	**25.0**	**25.0**	**25.0**	**25.0**	**0.0%**	**0.0%**	**0.0%**	**125.0**
			LA Factor		1.00	1.00	1.00	1.00				
		France	DDDA	1	25.0	25.0	25.0	25.0	0.0%	0.0%	0.0%	
		Germany	DDDA	1	25.0	25.0	25.0	25.0	0.0%	0.0%	0.0%	
		Spain	DDDA	1	25.0	25.0	25.0	25.0	0.0%	0.0%	0.0%	

Table S3. Production parameters from the sampled flocks.

Country	Number of Farms	Number of Poults Set-Up (Min – Max)	Mode* of Density in the Sampled Flock in kg/m² (Min – Max)	Avg Slaughter Age in Days (Min – Max)
**B**	20	7,093 (2997–13,000)	[51–55] ([46–50]–[56–60])	133 (96–147)
**E**	20	7799 (3000–21,794)	[≤ 40] ([≤40]–[≤40])	107 (74–140)
**H**	20	9,914 (4240–22,000)	[46–50] ([41–45]–[61–65])	101 (86–118)

**Total**	60	8,289<br>(2997–22000)	[≤40]<br>([≤ 40]–[61–65]))	113<br>(74–147)

Min = minimum, Max = maximum, Mode = most occurring answer from a drop down list with 10 levels, Avg = Average.

Table S3. bis. Number of poultry holdings in 2013 (other than broilers and laying hens) stratified by number of birds at the holding in country B, E and H.

Number of Birds on the Farm	Number of Poultry Holdings Other than Broilers and Laying Hens		
	Country B	Country E	Country H
**0**	9,040	13,170	4 480
**1–99**	990	4,790	2 530
**100–999**	210	1,470	40
**1000–2,999**	20	510	10
**3,000–4,999**	10	570	0
**5,000–9,999**	10	640	0
**10,000–49,999**	20	800	30
**50,000–99,999**	10	40	0
**≥100,000**	0	0	0
**Total**	10,310	7,090	22,000

*Data retrieved from Eurostat data explorer [49].

Table S4. $TIDDD_{Turkey}$ at flock level in country B, E and H.

Farm	Country		
	B	E	H
1	0.0	0.0	0.0
2	0.0	0.0	5.9
3	0.0	0.0	6.5
4	2.6	0.2	8.5
5	2.8	0.4	10.0
6	4.3	1.5	10.1
7	5.5	3.6	10.2
8	6.1	4.1	10.2
9	6.4	5.9	11.2
10	7.7	8.3	11.4
11	8.2	8.7	12.5
12	8.5	9.4	12.6
13	9.8	11.2	12.6
14	11.6	12.2	14.8
15	12.2	17.4	15.5
16	12.4	18.3	28.1
17	14.4	22.4	30.9
18	14.6	24.1	36.3
19	16.4	25.4	55.5
20	16.9	25.9	65.7

Table S5. Proportion (%) of the total amount of antimicrobials used (= total sum of $TIDDD_{turkey}$) and the number of treatments (n) at the 60 participating turkey flocks in country B, E and H (20 flocks/country), stratified by antimicrobial class, administration route and country.

Antimicrobial class	Water				Injection		Feed		Grand Total
	Country B	Country E	Country H	Total	Country H	Total	Country H	Total	
[1]ES Penicillins	3.1% (8)	6.0% (24)	24.2% (40)	33.2% (72)	- -	- -	- -	- -	33.2% (72)
Polymyxins	3.6% (5)	11.8% (18)	5.6% (8)	21.0% (31)	1.0% (4)	1.0% (4)	- -	- -	22.0% (35)
Fluoroquinolones	3.0% (7)	1.3% (3)	12.6% (18)	17.0% (28)	- -	- -	- -	- -	17.0% (28)
Macrolides	3.2% (11)	3.8% (13)	1.1% (4)	8.1% (28)	- -	- -	2.3% (5)	2.3% (5)	10.4% (33)
Tetracyclines	4.0% (7)	2.2% (6)	1.5% (4)	7.7% (17)	- -	- -	1.5% (7)	1.5% (7)	9.2% (24)
[2]BLS penicillins	3.2% (23)	- -	- -	3.2% (23)	- -	- -	- -	- -	3.2% (23)
[3]Trim/Sulfa	0.5% (2)	2.2% (4)	- -	2.7% (6)	- -	- -	- -	- -	2.7% (6)
[4]Linco/Spec	1.1% (2)	- -	- -	1.1% (2)	0.8% (4)	0.8% (4)	- -	- -	2.0% (6)
Aminoglycosides	0.3% (2)	- -	- -	0.3% (2)	- -	- -	- -	- -	0.3% (2)
Other Quinolones		0.04% (1)		0.04% (1)					
Grand Total	22.1% (67)	27.3% (69)	45.0% (74)	94.4% (210)	1.9% (8)	1.9% (8)	3.8% (12)	3.8% (12)	100% (230)

[1]ES penicillins = extended spectrum penicillins; [2]BLS penicillins = β-Lactamase-sensitive penicillins; [3]Trim/Sulfa = trimethoprim in combination with a sulfonamide; [4]Linco/Spec = lincomycin in combination with spectinomycin.

Table S6. Percentage of the total number of treatments within a country and in total presented per indication.

Indication for Treatment	Country B (n = 67)	Country E (n = 67)	Country H (n = 94)	Total (n = 230)
Intestinal disorders	65.7%	61.2%	56.4%	60.5%
Respiratory disorders	32.8%	14.9%	36.2%	28.9%
Colibacillosis	1.5%	16.4%	6.4%	7.9%
Locomotive disorders	-	4.5%	-	1.3%
General disorders	-	-	1.1%	0.4%
Non-specific disorders	-	1.5%	-	0.4%
Higher mortality rate	-	1.5%	-	0.4%

Table S7. Proportion of the total amount of antimicrobials used (= total sum of $\text{TIDDD}_{\text{turkey}}$) at the 60 participating turkey flocks in country B, E and H (20 flocks/country), stratified by antimicrobial class, administration route and country.

	**Week 1**	**Week 2**	**Week 3**	**Week 4**	**Week 5**	**Week 6**	**Week 7**	**Week 8**	**Week 9**	**Week 10**	**Week 11**	**Week 12**	**Week 13**	**Week 14**	**Week 15**	**Week 16**	**Week 17**	**Week 18**
Intestinal disorders (n = 138)	5.8%	10.1%	10.9%	8.7%	13.8%	10.1%	8.7%	10.1%	5.1%	3.6%	5.8%	1.4%	3.6%	0.7%	0.7%	0.7%	-	-
Respiratory disorders (n = 66)	6.1%	1.5%	4.5%	9.1%	1.5%	10.6%	7.6%	10.6%	13.6%	7.6%	6.1%	9.1%	3.0%	4.5%	-	-	1.5%	3.0%
Colibacillosis (n = 17)	11.8%	5.9%	-	-	29.4%	-	5.9%	11.8%	11.8%	5.9%	5.9%	-	-	-	-	11.8%	-	-
Locomotive disorder (n = 3)	-	-	-	-	-	-	-	-	66.7%	-	-	-	-	33.3%	-	-	-	-
Non-specific disorders (n = 1)	-	-	-	100.0%	-	-	-	-	-	-	-	-	-	-	-	-	-	-
General disorders (n = 1)	-	-	-	-	-	-	-	-	100.0%	-	-	-	-	-	-	-	-	-
higher mortality rate (n = 1)	100.0%	-	-	-	-	-	-	-	-	-	-	-	-	-	-	-	-	-

Table S8. Proportion of the total amount of antimicrobials used (= total sum of $TIDDD_{turkey}$) at the 60 participating turkey flocks in country B, E and H (20 flocks/country), stratified by antimicrobial class, administration route and country.

	**Week 1**	**Week 2**	**Week 3**	**Week 4**	**Week 5**	**Week 6**	**Week 7**	**Week 8**	**Week 9**	**Week 10**	**Week 11**	**Week 12**	**Week 13**	**Week 14**	**Week 15**	**Week 16**	**Week 17**	**Week 18**
[1]ES Penicillins (n = 72)	4.2%	13.9%	8.3%	11.1%	12.5%	13.9%	4.2%	8.3%	8.3%	5.6%	1.4%	1.4%	2.8%	1.4%	1.4%	1.4%	-	-
Polymyxins (n = 35)	5.7%	8.6%	2.9%	5.7%	17.1%	8.6%	14.3%	11.4%	2.9%	8.6%	5.7%	5.7%	-	-	-	2.9%	-	-
Fluoroquinolones (n = 28)	7.1%	-	10.7%	-	14.3%	17.9%	7.1%	21.4%	10.7%	7.1%	-	3.6%	-	-	-	-	-	-
Macrolides (n = 33)	12.1%	-	12.1%	18.2%	3.0%	-	6.1%	3.0%	12.1%	3.0%	6.1%	9.1%	3.0%	9.1%	-	-	3.0%	-
Tetracyclines (n = 24)	16.7%	4.2%	8.3%	4.2%	-	8.3%	8.3%	-	20.8%	-	12.5%	4.2%	-	4.2%	-	-	-	8.3%
[2]BLS penicillins (n = 23)	-	8.7%	8.7%	8.7%	4.3%	-	13.0%	21.7%	4.3%	-	13.0%	-	17.4%	-	-	-	-	-
[3]Trim/Sulfa (n = 5)	-	-	-	-	60.0%	-	-	-	20.0%	-	-	-	-	-	-	20.0%	-	-
[4]Linco/Spec (n = 6)	-	-	-	-	16.7%	16.7%	33.3%	16.7%	-	16.7%	-	-	-	-	-	-	-	-
Aminoglycosides (n = 2)	-	-	-	-	-	-	-	-	-	-	100.0%	-	-	-	-	-	-	-
Other Quinolones (n = 1)						100.0%												

[1]ES penicillins = extended spectrum penicillins; [2]BLS penicillins = β-Lactamase-sensitive penicillins; [3]Trim/Sulfa = trimethoprim in combination with a sulfonamide; [4]Linco/Spec = lincomycin in combination with spectinomycin.

Table S9. Correlations between the different indicators for quantifying antimicrobial usage from a Spearman's rank correlation test and corresponding *p*-values of a pairwise Wilcoxon signed rank test.

	TI_{DDDvet} (*p*-value)	$TI_{DDDturkey}$ (*p*-value)	$TI_{DDDturkey-national}$ (*p*-value)	TI_{DCDvet} (*p*-value)	$TI_{DCDturkey}$ (*p*-value)	TI_{UDD} (*p*-value)
TI_{DDDvet}	1[a,b]	0.98[b] ($p = 0.42$)**	0.97[b] ($p = 0.55$)**	1.00[b] ($p = 8.80e-9$)*	0.89[b] ($p = 4.70e-8$)*	0.95[b] ($p = 1.30e-7$)*
**$TI_{DDDturkey}$**	0.92[a] ($p = 0.02$)**	1[a,b]	0.99[b] ($p = 0.80$)**	0.97[b] ($p = 8.00e-10$)*	0.91[b] ($p = 4.30e-9$)*	0.94[b] ($p = 1.40e-8$)*
**$TI_{DDDturkey-national}$**	0.82[a] ($p = 0.02$)**	0.90[a] ($p = 0.90$)**	1[a,b]	0.95[b] ($p = 1.30e-9$)*	0.95[b] ($p = 7.40e-9$)*	0.93[b] ($p = 2.80e-8$)*
**TI_{DCDvet}**	0.98[a] ($p < 2.20e-16$)*	0.89[a] ($p < 2.20e-16$)*	0.82[a] ($p < 2.20e-16$)*	1[a,b]	0.88[b] ($p = 0.40$)**	0.95[b] ($p = 0.19e-2$)**
**$TI_{DCDturkey}$**	0.86[a] ($p < 2.00e-16$)*	0.98[a] ($p < 2.00e-16$)*	0.87[a] ($p < 2.00e-16$)*	0.85[a] ($p = 0.11e-2$)**	1[a,b]	0.85[b] ($p = 0.27e-1$)**
**TI_{UDD}**	0.49[a] ($p = 1.27e-5$)*	0.52[a] ($p = 0.05$)**	0.56[a] $p = 0.05$)**	0.45[a] ($p = 2.20e-16$)*	0.50[a] ($p = 2.20e-16$)*	1[a,b]

[a] correlation at treatment level, [b] correlation at flock level. * pairwise Wilcoxon signed rank test showed a significant difference between the two indicators, **pairwise Wilcoxon rank test did not show a significant difference between the two indicators. *p*-values of the Spearman's rank correlation test were all < 0.001.

Supplementary Materials Part B: additional information on data collection and data analysis

Uniform data collection on antimicrobial usage

Because of the big geographic area that was covered by the sampled farms within the whole EFFORT project, it was not possible to have each farm visited by the same researcher. Therefore, to reduce interview bias, each country had responsible samplers that received a training on the sampling protocols. This training was based on the results of a pilot study, that included 2 broilers farms in 2 countries and a questionnaire that was structured in a similar way. These broiler farms were also seen as a pilot study for the turkey farms within the project. The training was given before sampling started. After this training, the responsible samplers worked with their own team of researchers and veterinarians to execute the farm visits. When questions arose during sampling, the group of veterinarians that performed the pilot study were contacted for clarification.

Questionnaire

The questionnaire included here is an abbreviated version. Only parts that are relevant for the data discussed in the publication are shown.

I. Confidentiality statement

Farmer: I declare that I have read the leaflet with information about this study and I approve that all data collected on the herd will be used anonymously for further analysis and publications by the EFFORT consortium.

☐ The researchers can contact me within 1 month from the date below if data would be missing.

☐ The researchers can contact me for additional research projects

Date: …………………………………. Name: ………………………………………………………………………………

Signature:

……

…………………

Researcher: I declare that I have taken all necessary measures to inform the farmer about the purpose and organisation of the Effort research project and that the data collected on this herd will be processed anonymously and will only be used for further analysis and publication within the EFFORT consortium.

Date: …………………………………….. Name:

………………………………………………………………………………………

Signature:

……

…………………

**Date of completion of the questionnaire (dd/mm/yyyy)**	……… / ……… / ……………
**Farm code**	

II. General information

2.1. Administrative details turkey manager

*** Name + first name**	
**Telephone nr.**	
*** Mobile phone nr.**	
*** Email address**	

2.2. Administrative details turkey farm

**Company name**	
*** Address**	Street: nr.:
	Postal code: City:
	Country:
*** GPS coordinates**	Longitude: Latitude:
	Street: nr.:

* Correspondence address (only if different from farm address)	Postal code:	City:
	Country:	

2.3. Administrative details herd veterinarian/veterinary practice

Name + first name	
Telephone nr.	
Mobile phone nr.	
Email address	

* Mandatory information. Below, answers on all questions are mandatory.

III. Farm characteristics

1. How many turkeys in total are currently present on the farm?..
2. Are there, besides turkeys, other animals present at the farm?
   No
   Yes, which?

How many people are working on the turkey farm in total? ....

Characteristics of sampled house

1. How many turkeys were set-up in the current round in the sampled house?
2. What was the average weight of the turkeys at set-up?
3. How many turkeys are currently present in the sampled house?
4. How old are the turkeys currently present in the sampled house?
5. What is the weight of the female turkeys currently present in the sampled house?
6. What is the weight of the male turkeys currently present in the sampled house?
7. Expected age at delivery to the slaughterhouse?

Group 1:...

Group 2:...

Other groups:...

IV. Antimicrobial usage

Group treatments

Treatment Type	Number of Treated Animals	Product Name	Active Substance	Concentration	Used Dose	Administration Route	Treatment Duration (Days)	Weight at Treatment (kg)	Age at Treatment (Days)	Indication
Standard<br>Non-standard<br>Coccidiostatic						Medicated feed – feed mixed at herd<br>Water<br>Spray<br>Injection				Respiration<br>Intestines<br>Locomotion<br>Nervous disorders<br>Dermatology<br>Eye<br>General disorder<br>Colibacillosis<br>Omphalitis<br>Non-specific mortality

References

1. World Health Organisation (WHO) *Critically Important Antimicrobials for Human Medicine 5th Revision 2016 Ranking of medically important antimicrobials for risk management of antimicrobial resistance due to non-human use*; 2016;
2. World organisation for animal health (OIE) *OIE list of antimicrobial agents of veterinary importance*; 2018;
3. Eurostat Eurostat - Data Explorer Available online: http://appsso.eurostat.ec.europa.eu/nui/show.do (accessed on Aug 6, 2021).
